# Simplifying the Diagnosis of Pediatric Nystagmus with Fundus Photography

**DOI:** 10.3390/children12020211

**Published:** 2025-02-11

**Authors:** Noa Cohen-Sinai, Inbal Man Peles, Basel Obied, Noa Netzer, Noa Hadar, Alon Zahavi, Nitza Goldenberg-Cohen

**Affiliations:** 1 Department of Ophthalmology, Bnai Zion Center, Haifa 3339419, Israel; noabarsinai@gmail.com (N.C.S.); pelesman@gmail.com (I.M.P.); basel.obied01@gmail.com (B.O.); merkeln@gmail.com (N.N.); noahadar78@gmail.com (N.H.); 2 Faculty of Medicine, Tel Aviv University, Tel Aviv 6997801, Israel; alonzahavi@gmail.com; 3 Department of Ophthalmology and Laboratory of Eye Research, Felsenstein Medical Research Center, Rabin Medical Center—Beilinson Hospital, Petach Tikva 4941492, Israel; 4 The Krieger Eye Research Laboratory, Faculty of Medicine, Technion, Israel Institute of Technology, Haifa 3109601, Israel

**Keywords:** nystagmus, fundus photography, retinal imaging, children

## Abstract

Background/Objectives: To simplify diagnosing congenital and acquired nystagmus using fundus photographs. Methods: A retrospective study included patients with congenital or childhood-acquired nystagmus examined at a hospital-based ophthalmology clinic (September 2020–September 2023) with fundus photos taken. Exclusions were for incomplete data or low-quality images. Demographics, aetiology, orthoptic measurements, and ophthalmologic and neurological exams were reviewed. Two independent physicians graded fundus photos based on amplitude (distance between “ghost” images), the number of images visible, and the direction of nystagmus. Severity was rated on a 0–3 scale using qualitative and quantitative methods. Photographic findings were compared to clinical data, and statistical analysis used Mann-Whitney tests. Results: A total of 53 eyes from 29 patients (16 females, 13 males; mean age 12.5 years, range 3–65) were studied: 25 with binocular nystagmus and 3 with monocular nystagmus. Diagnoses included congenital (*n* = 15), latent-manifest (*n* = 3), neurologically associated (*n* = 2), and idiopathic (*n* = 9). Types observed were vertical (*n* = 5), horizontal (*n* = 23), rotatory (*n* = 10), and multidirectional (*n* = 15). Visual acuity ranged from 20/20 to no light perception. Fundus photos correlated with clinical diagnoses, aiding qualitative assessment of direction and amplitude and mitigating eye movement effects for clearer retinal detail visualization. Conclusions: Fundus photography effectively captures nystagmus characteristics and retinal details, even in young children, despite continuous eye movements. Integrating fundus cameras into routine practice may enhance nystagmus diagnosis and management, improving patient outcomes.

## 1. Introduction

Nystagmus is an involuntary, rhythmic oscillation of the eyes, characterized by alternating slow and fast phases of movement [1]. Each type of nystagmus exhibits unique features, which are categorized based on direction, movement plane, amplitude, frequency, speed, and symmetry [1]. The eye movements can be horizontal, vertical, torsional, or a combination of these. The continuous motion often impairs visual acuity (VA) by causing excessive image displacement on the retina, reducing the duration the fovea can maintain fixation on objects [2,3,4].

Nystagmus is typically categorized into two types: infantile nystagmus, which generally manifests within the first 3 to 6 months of life, and acquired nystagmus, which develops later. The most common nystagmus type in children is infantile, which can be idiopathic or associated with ocular, neurologic, and systemic disease [5]. Although the majority of the infantile nystagmus cases are idiopathic, others are associated with conditions such as albinism, retinal disease, low vision, or early visual deprivation caused by factors like congenital cataracts, optic nerve hypoplasia, retinal dystrophies, or as part of neurological syndromes or other neurological disorders [2,6,7]. Differentiating between types of nystagmus is crucial due to its potential association with various underlying diseases, some of which may be life-threatening [5]. This distinction requires considering both the onset of the nystagmus and its waveform characteristics [5].

The assessment and documentation of nystagmus are challenging and often difficult to perform in children with limited cooperation. Diagnostic accuracy depends on precise observation and identification of eye movement [8]. Several tools are available for the evaluation of nystagmoid eye movements, such as Frenzel glasses, electronystagmography, and infrared video nystagmography. Due to limitations with current assessment modalities, efforts have been made to develop alternative methods for nystagmus evaluation.

The aim of this study is to simplify nystagmus diagnosis in the outpatient clinic by using widely available non-dilated fundus cameras to enhance diagnostic accuracy and examination quality, particularly in challenging cases, such as children with developmental delays.

## 2. Materials and Methods

**Figure 1:**
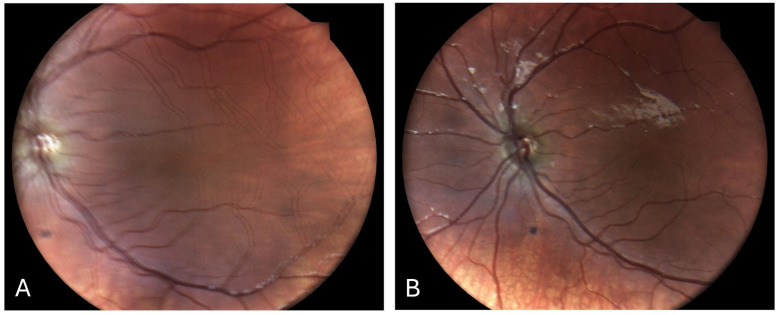
Left eye fundus photography of a patient with congenital horizontal nystagmus, taken before and after a modified Kestenbaum procedure performed to correct abnormal head position. (**A**) A ghost image, indicating small-amplitude horizontal nystagmus. (**B**) A single image, indicating the resolution of the nystagmus, consistent with the clinical findings.

A retrospective study design was used. Medical records of all patients with congenital or acquired nystagmus examined at the hospital outpatient clinic between September 2020 and 2023 who underwent fundus photography were reviewed (Figure 1). Data collection encompassed patient demographics, orthoptic measurements, ophthalmologic and neurological examination findings, and fundus photographs. Exclusion criteria were the insufficient documentation of eye movements or insufficient quality of fundus photography. All patients had nystagmus on clinical eye exam in at least one eye.

**Figure 2:**
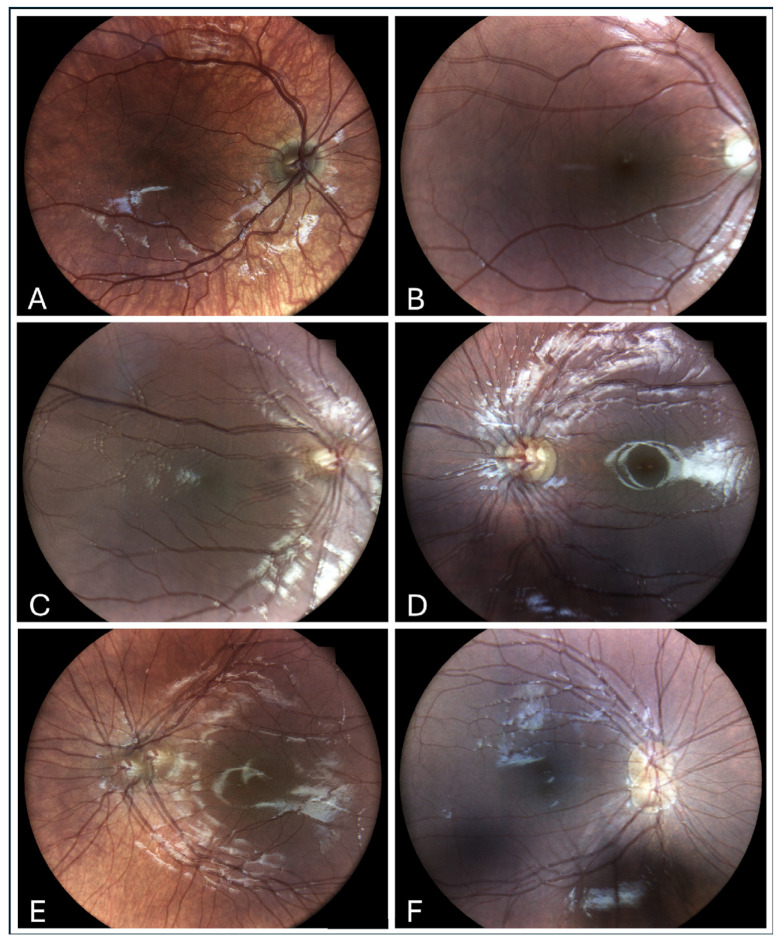
Typical fundus photos showing no double image (**A**), grade 1 rotatory nystagmus (**B**), grade 2 horizontal nystagmus with a vertical component (**C**), grade 3 horizontal nystagmus (**D**), grade 3 horizontal nystagmus with a vertical component (**E**), and grade 3 vertical nystagmus (**F**).

Fundus photographs were captured using the CenterVue non-mydriatic fundus camera DSplus (CenterVue S.p.A, Padova, Italy). Each photo was graded independently by two physicians: one performed a quantitative assessment by measuring pixel displacement between identical anatomical landmarks, such as blood vessel bifurcations on the “ghost” images. The other physician conducted a qualitative evaluation based on the overall appearance of the displaced images. Both grading methods used a scale from 0 to 3, where 0 indicated no visible nystagmus and 3 represented large amplitude nystagmus (Figure 2). The quality assessment also considered the number of images observed and their orientation to determine the direction of the nystagmus. The photographic grading results were then compared with each other and correlated with the clinical examination findings.

All calculations and statistical analyses were performed using GraphPad Prism software (version 10.2) using a Mann Whitney non-parametric test. Differences in samples between the two groups were assessed using the *t*-test. A *p*-value of less than 0.05 (two-tailed) was considered statistically significant.

Data collection and analysis adhered to the Declaration of Helsinki for research involving human participants. The study was approved by the local Institutional Review Board (IRB No. 0012-23).

## 3. Results

A total of 53 eyes with nystagmus from 29 patients were included in the study. Three patients were excluded, two due to insufficient clinical examination documentation and one due to poor photo quality caused by lack of cooperation. Additionally, one patient had a low-quality image in only one eye due to poor cooperation, resulting in the exclusion of that eye alone.

Patients’ demographic data are shown in Table 1. All patients had had nystagmus since childhood, either congenital or acquired. Table 2 presents the etiologies, direction, and amplitude of the nystagmus as diagnosed through clinical examination and fundus photo analysis. The mean VA was 0.56 log MAR, ranging from no light perception to 0.0 log MAR with both eyes.

Diagnosis of nystagmus could be easily achieved in 64.2% (34 eyes) of the cases by qualitative assessment of the fundus photos and in 66.03% (35 eyes) using quantitative measurements of the photographs.

The fundus photos allowed for the estimation of changes in strabismus over time during repeated visits, as well as the assessment of treatment effects, such as strabismus surgery or systemic therapy for underlying conditions. We found a correlation between reduced nystagmus and improved VA following modified Kestenbaum surgery in two children. The majority of the patients had a single set of fundus photos, while seven patients had repeated photos over years of follow-up with a mean follow-up of 25.2 months. Six of them showed no improvement, while one child underwent a modified Kestenbaum strabismus surgery and had improved, demonstrating reduced nystagmus frequency and amplitude. The seven patients who had sequential fundus photography on subsequent visits underwent an average of 4.3 imaging sessions. In four cases, nystagmus was consistently present in all photographs, while in the remaining three cases, nystagmus appeared in only one of the series of images. Of these three patients, paroxysmal nystagmus explained the intermittent nature in two cases, while one patient had nystagmus that was evident before surgery but resolved following a modified Kestenbaum procedure (Figure 1).

Fundus photography qualitative assessment in eyes that showed double or triple “ghost” images matched the clinical direction in 76.5% and amplitude in 61.8%. Quantitative assessment matched the clinical direction in 79.4% and amplitude in 64.7%. Additionally, the qualitative and quantitative assessments were consistent with each other in 70.6% of cases regarding direction and in 73.5% regarding amplitude.

Among the eyes in which no match was found (seven eyes, 20.5%), a rotary component was detected in the photographs in half of the cases, which had not been identified during the clinical examination.

In four out of six eyes with latent nystagmus (66.7%), no double ghost images were observed in fundus photography. In all patients with latent nystagmus, at least one eye showed no double images in the photographs.

### 3.1. Case 1

An 8-year-old girl had been followed by the ophthalmology outpatient clinic for bilateral congenital opsoclonus. She first presented to the clinic at 10 weeks old due to multidirectional conjugate eye movements with horizontal, vertical, and torsional components. The saccades were rapid and involuntary, with no inter-saccadic interval, null point, or rhythmic pattern, and they persisted during sleep. The saccades were also enhanced by visual fixation.

The patient had a normal pregnancy, growth, and development, and was breastfed. However, three years prior to her pregnancy the mother had been diagnosed with glioblastoma, which was resected. She was also treated at that time with adjuvant radiation and temozolomide chemotherapy, and a small residual lesion in her left frontal lobe remained stable. She died five years later.

Due to the early presentation of opsoclonus at 10 weeks, the patient underwent an extensive workup that included a thorough neurological examination, visual evoked potential testing, electroretinography, magnetic resonance imaging from brain to pelvis, and an abdominal ultrasound. Urine tests for vanillylmandelic acid and homovanillic acid, as well as blood tests for inflammatory markers and paraneoplastic antibodies, including anti-Ri, anti-Hu, anti-Yo, anti-Ma-1-2, and anti-GAD, were all within normal limits.

Given the negative findings and the maternal history, a presumptive diagnosis of paraneoplastic opsoclonus was made, hypothesizing that maternal exposure to brain tissue after tumor resection and potential antibody transfer during pregnancy could be the cause. Breastfeeding was discontinued as a precaution, but the opsoclonus persisted.

During her 8-year follow-up, the patient remained stable, with a best-corrected VA of 6/30 (20/100) for distance and J2 for near vision with both eyes open. Her medical history was otherwise unremarkable, and her growth and development were normal.

The patient exhibited rapid, small-amplitude, conjugate eye movements in rotary, vertical, and horizontal directions, without a slow phase or inter-saccadic interval, consistent with opsoclonus. There was no null point, and her head posture was inconsistent, alternating between right head tilt, right head turn, and chin up, with both eyes open. There was no improvement within the follow-up period.

Despite decreased VA, other optic nerve functions, including pupillary reactions, color vision, and confrontational visual fields, were normal. Ocular motility was full. The child was orthophoric without strabismus. Although the rapid eye movements made slit-lamp examination challenging, the anterior segments and intraocular pressure were normal. Fundus examination was even more difficult, demonstrating an enlarged cup-to-disc ratio with a normal posterior pole appearance.

**Figure 3:**
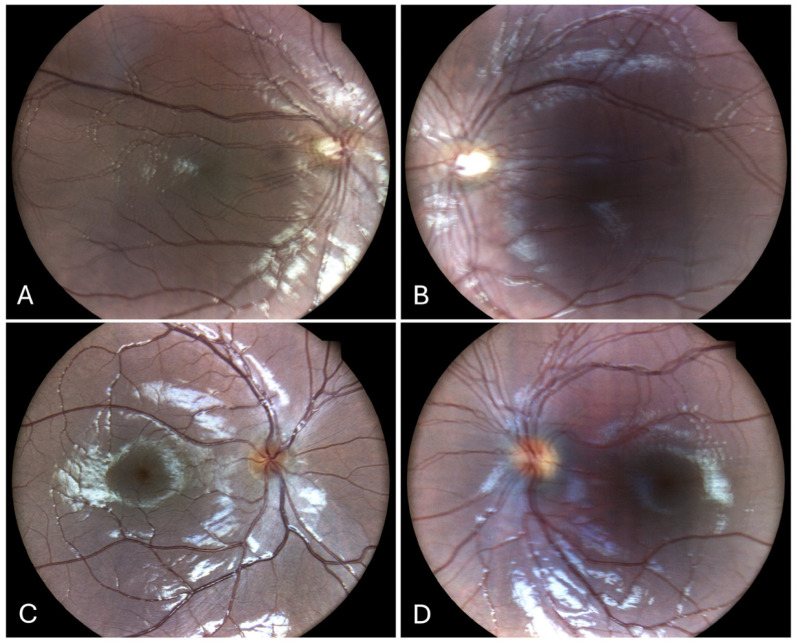
Fundus photos of Case 1 (**A**,**B**) showing double images with small-to-medium amplitude and both horizontal and vertical components in both eyes, consistent with clinical findings. The macula was normal. Blinded grading of the photographs yielded scores of 2 for the right eye and 3 for the left eye. Case 2 (**C**,**D**). 3C demonstrated a normal fundus with a single image. The left eye in image 3D displayed a normal fundus but duplicated photos, suggesting a small amplitude horizontal nystagmus, which corresponds to the clinical finding of latent nystagmus when the fellow eye is occluded.

Repeated fundus photographs (Figure 3A,B) revealed double images with small-to-medium amplitude and both horizontal and vertical components in both eyes, consistent with clinical findings. The macula was normal. Blinded grading of the photographs yielded scores of 2 for the right eye and 3 for the left eye.

### 3.2. Case 2

A 4-year-old boy underwent evaluation for strabismus. He was born at 32 weeks gestation, weighing 2280 g. There was no family history of strabismus. On examination, he had mild amblyopia in the right eye with a VA of 20/40 in the right and 20/30 in the left eye. The orthoptic exam showed 35 prism diopters of esotropia in the primary position with a V pattern (orthophoric in up gaze). The cycloplegic refraction was +1.75 D in both eyes. Ocular motility testing revealed a bilateral abduction deficit and bilateral dissociated vertical deviation. He also exhibited a small amplitude, horizontal latent nystagmus, which appeared when either eye was covered. He received treatment for amblyopia with atropine drops in the left eye, resulting in improved vision of 20/30 in the right eye. Slit-lamp ophthalmoscopic examination revealed normal anterior segments and fundus. Fundus photos were taken before and after strabismus surgery for bilateral medial rectus recession and inferior oblique myectomy to correct the V pattern esotropia.

After surgery, he was orthoptic in primary gaze for far, with a residual 8PD for near. Fundus photography of the right eye (Figure 3C) demonstrated a normal fundus with a single image. However, the left eye image (Figure 3D) displayed a normal fundus but duplicated photos, suggesting a small amplitude horizontal nystagmus, which corresponds to the clinical finding of latent nystagmus when the fellow eye is occluded.

## 4. Discussion

Here, we present a straightforward platform for assessing various types of nystagmus. Fundus photography does not only provide a detailed examination of the posterior segment but also enables nystagmus grading and facilitates follow-up during observation and treatment. This approach helps in assessing anatomical disorders of the macula and retina, overcoming the challenge of examining a crying child by indirect ophthalmoscopy, and potentially negates the need for evaluation under anesthesia. Nystagmus is a challenging disorder due to its diverse etiologies, ranging from ocular anatomical disturbances to severe neurological conditions and systemic disorders. Additionally, nystagmus can negatively affect VA and is often difficult to treat.

The prevalence of infantile nystagmus syndrome has been reported as 1 in 821 [9]. In infantile nystagmus, oscillations are rare, whereas patients with acquired nystagmus commonly complain of oscillations that may be associated with imbalance and dizziness [6,7]. The incidence of all types of pediatric nystagmus was previously reported as 6.72 per 100,000 in patients younger than 19 years, with most infantile nystagmus patients maintaining good vision but often presenting with developmental delay, strabismus, or amblyopia [9].

Life-threatening forms of nystagmus include opsoclonus as a paraneoplastic sign, mainly for neuroblastoma, and vertical (downbeat or upbeat) nystagmus resulting from cerebellar or brainstem lesions [10,11]. See-saw nystagmus is pathognomonic for lesions in the rostral midbrain and suprasellar region, with possible involvement of the optic chiasm and extension into the third ventricle [12,13]. Periodic alternating nystagmus can result from cerebellar compression (e.g., Arnold Chiari malformation), multiple sclerosis, or encephalitis [4,14]. Neurological conditions should be suspected when the nystagmus is asymmetrical or unilateral. Monocular nystagmus has been reported as an initial presenting sign of chiasmal glioma in young children [15]. A thorough evaluation, including electrophysiological testing, laboratory assessments, and imaging, is essential to rule out underlying ocular or systemic pathologies [2,4].

Although electronystagmography is a quantitative method for recording eye movements, it is seldom used in outpatient clinics and cannot reliably capture torsional components. VNG is considered the gold standard for evaluating nystagmus, as it assesses all three dimensions of ocular movements—horizontal, vertical, and torsional [16]. In a typical VNG test, the patient wears goggles equipped with an infrared camera to track pupil movements, with specialized software analyzing nystagmus characteristics [17]. While VNG can include various subsets and is usually performed by an audiologist, its adoption is limited by high costs and the need for an office-based setup with a trained specialist [17]. Sefein et al. designed modified goggles with a built-in camera and software, creating a portable device for diagnosing horizontal nystagmus [18], while Friedrich et al. introduced a smartphone-based video nystagmography system using a neural network for nystagmus detection and data extraction [19].

Examining pediatric patients with nystagmus is difficult due to limited cooperation and attention, rapid eye movements, light-induced blur, and frequent associated disorders. Moreover, fundus examination, even with a well-dilated pupil, can be particularly challenging in children, and even more in the presence of nystagmus. Non-mydriatic fundus photography offers a comfortable, non-invasive alternative that can reduce stress and irritation, as many children are already familiar with digital devices. The process of capturing still images also allows clinicians to overcome issues such as constant eye movement and patient resistance, providing higher-quality images than are often obtained via indirect ophthalmoscopy.

Eye movement recordings and microperimetry have been widely used to measure and quantify nystagmus [20,21,22]. However, we found no prior studies in the literature that utilized fundus photographs for similar quantitative assessments. Spectral-domain optical coherence tomography (SD-OCT), which includes fundus imaging, has been shown to effectively capture macular morphology in patients with congenital nystagmus [23]. While SD-OCT does not directly assess nystagmus, its ability to obtain high-resolution images despite involuntary eye movements suggests that fundus imaging techniques can be successfully applied to patients with nystagmus. The increased scanning speed of SD-OCT (50 to 100 times faster than previous technologies) has been a key factor in overcoming motion-related artifacts [23]. Additionally, the feasibility of non-mydriatic fundus photography in children has been demonstrated [24]. Although this study did not specifically focus on nystagmus, it showed that fundus photography is a practical and time-efficient method for acquiring high-quality images in children over the age of three.

Given the absence of an established grading system for assessing nystagmus from fundus photographs, we developed a practical approach based on measuring the pixel distance between ghost images. This method provides a simple and reproducible way to quantify nystagmus amplitude. We anticipate that artificial intelligence will further refine and enhance the accuracy of these measurements in the future.

In this study, we found that fundus images alone can facilitate the examination of not only the optic disc and macula but also the direction and amplitude of the nystagmus. The etiologies detected in our study included latent nystagmus (10.3%), congenital nystagmus (51.7%), neurologically associated nystagmus (6.9%), and idiopathic nystagmus (31%). Although our cohort included both children and adults, all but three of the adults had congenital nystagmus, and the acquired cases occurred in childhood. According to Nash et al. [9], the main types of pediatric nystagmus are retinal/optic nerve disease (32.4%), idiopathic/congenital motor nystagmus (31.0%), manifest latent or latent nystagmus (24.0%), and other less common etiologies. In their series, 80% presented with 20/40 or better vision in at least one eye, underscoring the need for accurate characterization and follow-up.

A clear view of the macula is essential, as some nystagmus are due to macular hypoplasia. In children with albinism, associations have been noted among pendular or jerk nystagmus, foveal hypoplasia grade, and VA [25]. Current treatment options for infantile nystagmus syndrome focus on optimizing vision by reducing nystagmus intensity and correcting anomalous head postures and associated strabismus [5]. Pharmacological treatment options such as gabapentin and memantine have been introduced into clinical practice [5]. In patients with torticollis, eye muscle surgery may shift the nystagmus null zone into primary position. Because an accurate diagnosis is crucial, we suggest using fundus photographs—potentially alongside genetic testing—to refine the characterization of nystagmus and to monitor treatment outcomes. Recent advances in pediatric retinal imaging, including objective calculations based on pixel shifts, can add precision to our current approaches.

Non-mydriatic fundus cameras have already proven efficient and reliable in diagnosing various ocular and systemic conditions in diverse settings. Their use in emergency departments has been advanced by pioneers such as Drs Newman and Biouss, demonstrating the transformative potential of this technology [26,27,28,29,30]. Their studies suggest that digital retinal photography can effectively replace direct ophthalmoscopy, which is often challenging for non-ophthalmologists [26,27,28,31]. Studies also show that non-mydriatic imaging exhibits high sensitivity and specificity compared with standard clinical ophthalmology examinations [27,28,29,30]. For example, the Fundus Photography vs. Ophthalmoscopy Trial Outcomes in the Emergency Department (FOTO-ED) study revealed that emergency department providers frequently missed critical findings with direct ophthalmoscopy, whereas non-mydriatic imaging offered a reliable alternative [29]. Looking ahead, artificial intelligence and deep learning technologies promise to enhance the analysis of fundus photographs, provide objective measurements, improve diagnostic accuracy, and potentially revolutionize follow-up care and patient outcomes in pediatric ocular pathologies and neuro-ophthalmology.

Assessing nystagmus via fundus photography has both advantages and limitations. In eyes with double or triple ghost images, clinical diagnosis and qualitative assessment agreed in 76.5% of cases for direction and 61.8% for amplitude; quantitative assessment showed similar concordance. Discrepancies were often due to overestimation of amplitude in qualitative evaluations or misclassifications of direction as “mixed”. Small-amplitude nystagmus proved difficult to detect on still images, as subtle oscillations produce minimal artifacts. Latent or intermittent nystagmus might not appear in a single photograph but can be captured in at least one image over multiple sessions. Rotatory components were sometimes clinically overestimated but not observed in fundus images, suggesting potential overdiagnosis in standard examinations. Fundus photography may indeed underestimate the severity of nystagmus compared to clinical examination due to the relatively short camera exposure time, which can fail to capture rapid eye movements. However, we consider this one of the advantages of fundus photography, as it allows for a clear and detailed static image of the fundus, which is often superior to what can be observed with direct or indirect ophthalmoscopy. The static nature of the image also provides qualitative insight into the impact of nystagmus on foveation time. Unlike video-based assessments that capture continuous movement, fundus photography offers a snapshot that reflects the degree of retinal image displacement, which can be useful for assessing how nystagmus affects visual fixation. The non-mydriatic fundus camera used in our study has an exposure time in the range of milliseconds. This brief exposure helps minimize motion blur, allowing for the capture of fine retinal details even in patients with involuntary eye movements. While this limitation may lead to underestimation of nystagmus severity, it also enables accurate visualization of retinal structures, facilitating clinical evaluation and long-term follow-up.

While fundus photography has limitations in capturing the rhythmic patterns of nystagmus, the examiner can observe the eye movements on the screen in real-time during the examination. This allows for direct visualization of nystagmus, and the findings can be documented in the patient’s record. The combination of fundus photography and real-time observation enhances the diagnostic process and is an added advantage of using the fundus camera. Additionally, the CenterVue DSplus fundus camera used in the study provides an option to view external eye movements alongside retinal motion in a small window, enabling a more comprehensive assessment of nystagmus characteristics.

Another key finding was the tendency for clinicians to overestimate amplitude, likely due to subjective interpretation. Fundus photography offers a more standardized view that may reduce such overestimations and refine nystagmus assessment.

Two independent ophthalmologists evaluated the nystagmus direction and amplitude in our fundus images. Although their quantitative measurements differed significantly, their qualitative assessments were consistent. Consequently, we recommend using non-parametric evaluation of the retinal photos, as shown in Figure 1, to achieve better correlation with clinical findings. By integrating repeated fundus photography with clinical examination, clinicians can obtain a more accurate and comprehensive understanding of nystagmus characteristics, thereby improving both diagnosis and management.

## 5. Conclusions

This study highlights the utility of fundus photography as a valuable tool for assessing and documenting pediatric nystagmus. Our findings demonstrate that fundus imaging provides an effective method for evaluating the direction and amplitude of nystagmus while simultaneously capturing critical retinal details, even in young and uncooperative patients. The strong correlation between photographic and clinical assessments underscores the potential of fundus photography to complement existing diagnostic techniques. By incorporating this approach into routine clinical practice, ophthalmologists may enhance diagnostic accuracy, streamline patient monitoring, and facilitate objective tracking of disease progression and treatment efficacy. Further research integrating artificial intelligence and quantitative image analysis may further refine nystagmus characterization and improve patient outcomes.

## Figures and Tables

**Table 1:** Patient demographics in 29 patients (53 eyes).

Parameter	Number
Total number of patients	29
Adults (>18 years)	5
Children (<18 years)	24
Age (y), average (range)	
Adults	34.5 (23–65)
Children	7.9 (3–16)
Total	12.5 (3–65)
Sex, *n* (%)	
Male	13 (44.8%)
Female	16 (55.2%)
Visual acuity (logMar), mean (range)	0.56 (NLP–0.0)
Associated conditions, *n* (%)	
Albinism	3 (10.3%)
Prematurity	3 (10.3%)
Retinal disease	3 (10.3%)
Cataract	3 (10.3%)
Other systemic syndrome	3 (10.3%)

**Table 2:** Characteristics of nystagmus etiologies, direction, and amplitude as diagnosed through clinical examination and fundus photo analysis.

Parameter	N				
Nystagmus etiology, *n* (%)					
Congenital	15 (51.7%)				
Latent manifest	3 (10.3%)				
Neurological associated	2 (6.9%)				
Unknown	9 (31%)				
Direction of nystagmus (53 eyes total)	Horizontal	Vertical	Rotary	Combined	Undetected
Clinical examination	23	5	10	15	0
Fundus photo quantitative analysis	17	3	4	11	18
Fundus photo general appearance	16	9	1	8	19
Grading of nystagmus amplitude	Not detected	1 (small)	2 (medium)	3 (large)	
Clinical examination	0	4	24	25	
Fundus photo quantitative analysis	19	5	12	17	
Fundus photo qualitative assessment	19	4	7	23	

## Data Availability

The original contributions presented in this study are included in the article. Further inquiries can be directed to the corresponding author.

## Abbreviations

The following abbreviation is used in this manuscript:

VA	Visual acuity
